# National Approaches to Monitoring Population Salt Intake: A Trade-Off between Accuracy and Practicality?

**DOI:** 10.1371/journal.pone.0046727

**Published:** 2012-10-17

**Authors:** Corinna Hawkes, Jacqui Webster

**Affiliations:** 1 World Cancer Research Fund International, London, United Kingdom; 2 Independent Food Policy and Public Health Specialist, Liverpool, United Kingdom; 3 The George Institute for Global Health, University of Sydney, Sydney, Australia; Lausanne University Hospital and University of Lausanne, Switzerland

## Abstract

**Aims:**

There is strong evidence that diets high in salt are bad for health and that salt reduction strategies are cost effective. However, whilst it is clear that most people are eating too much salt, obtaining an accurate assessment of population salt intake is not straightforward, particularly in resource poor settings. The objective of this study is to identify what approaches governments are taking to monitoring salt intake, with the ultimate goal of identifying what actions are needed to address challenges to monitoring salt intake, especially in low and middle-income countries.

**Methods and Results:**

A written survey was issued to governments to establish the details of their monitoring methods. Of the 30 countries that reported conducting formal government salt monitoring activities, 73% were high income countries. Less than half of the 30 countries, used the most accurate assessment of salt through 24 hour urine, and only two of these were developing countries. The remainder mainly relied on estimates through dietary surveys.

**Conclusions:**

The study identified a strong need to establish more practical ways of assessing salt intake as well as technical support and advice to ensure that low and middle income countries can implement salt monitoring activities effectively.

## Introduction

This paper reviews the experiences of national government efforts to monitor salt intake around the world. The objective is to identify and review the different approaches that governments are taking to monitoring intake, with the ultimate goal of understanding what actions are needed to address some of the challenges posed, especially in low and middle-income countries where resources are often more limited.

There is clear evidence that eating too much sodium, mainly in the form of salt (sodium chloride), has adverse implications for health. [1] Sodium intake is associated with elevated blood pressure, which is a leading risk for cardiovascular disease, a major risk factor for premature death globally. The World Health Organization (WHO) estimates that the prevalence of elevated blood pressure levels is on average slightly higher in low and middle-income countries. [2]

There is also evidence that reducing population salt intakes will reduce the burden of disease. [3], [4] It has been estimated that reducing salt intake by 6 g/day would reduce stroke death s by 23–25% and deaths from Coronary Heart Disease (CHD) by 16–19%. [5] Salt reduction activities are, moreover, estimated to be highly cost effective. [6] As a result, leading experts recommend that governments make salt reduction a top health priority, [7], [8] a recommendation also made by delegates at the 2011 United Nations High-level Meeting on the Prevention and Control of Non Communicable Diseases (NCDs).

Many governments are now implementing salt reduction strategies, which typically include targets for the food industry to reformulate processed food products, mass communication campaigns, and monitoring. [9], [10] Monitoring of salt intake is undertaken to determine the extent of the problem, identify where efforts to reduce salt intake should be targeted, and demonstrate the impact of strategies. [9] [11]

Yet monitoring salt intake is not straightforward. The main methods available are 24 hour urine collection, spot urine collection and dietary surveys. 24 hour urine analysis is considered the “gold-standard” from the perspective of scientific accuracy, primarily because of the problems of underestimation with dietary surveys and spot urine methods. A 24 hour period is necessary to capture the fluctuations in sodium excretion that occur each day, however this method does not account for electrolyte loss other than via the kidneys, and therefore will tend to slightly underestimate the true intake, particularly in very hot countries. What's more collecting 24 hour urine samples for a representative sample of the population is challenging and resource-intensive [12], [13]. Rather than collecting urine over 24 hours, spot urine collection means just taking a single sample at a certain time of the day, from which the sodium level can be tested and calculations done to establish what that would equate to over 24 hours. However the result is likely to differ depending on what time of the day the spot sample is taken and calculations based on this method are therefore likely to produce unreliable results. Dietary surveys usually involve questionnaires to understand exactly what people have eaten or how often certain foods have been eaten. This information can then be used in conjunction with food composition tables to assess sodium intakes. However, numerous studies show that people tend to under-report, so again the results are likely to be underestimations. Dietary surveys and spot urine measurements are considered less resource-intensive, but there are still conflicting perspectives about the extent to which these methods are able to produce reliable estimates [14], [15], [16], [17]

## Methods

A written survey targeted at countries believed to be taking some form of action on salt reduction was commissioned by Health Canada to inform a joint Government of Canada/WHO technical consultation on salt monitoring. Conducted between August and October 2010, the survey involved five core steps.

First, the development of the survey questionnaire. Nine questions were asked to identify monitoring methods (Q1–4) and experiences of implementing these methods (Q5–8), including perceptions of whether implementation had been successful, the barriers and constraints faced, the factors considered essential for success and lessons learned (Q5–8), plus one further question requesting results of monitoring where available (Q9) (see Supplement S1). Recipients were asked to complete the questionnaire in written form using free-style text.

Second, countries with salt monitoring activities were identified. The following sources were used: (i) recent reviews of existing salt reduction activities; [9], [18] (ii) a list provided by the WHO of low, lower-middle and middle income countries reported to have undertaken some form of salt monitoring ; (iii) the participant list of a WHO Technical Meeting on Salt Reduction in London in 2010; (iv) the membership list of the European Salt Action Network. [19] (v) a database of national activities compiled by the World Action on Salt and Health (WASH) [20].

Third, country contacts were identified. Contacts were obtained through the above sources and through the author's own networks. Where government contacts could not be identified, representatives from civil society - non-governmental organisations (NGO) and/or research institutions – were sought. Countries were included in the survey if the government or civil society organisations were conducting salt monitoring activities either independently or as part of broader food or health surveillance initiatives. Fourth, the survey was then emailed as a letter attachment to the contacts with a deadline for response.

Fifth, the results were analysed. This took two forms. Answers to Q1–4 on monitoring methods were tabulated by method (24 hour urine, spot urine, dietary survey) accompanied with details of the specific methodology used (type of survey, scale, age covered, survey frequency), plus the context of monitoring and future monitoring plans. These were then sorted according to national income groups [21] in order that the results could be analysed according to high, upper-middle, lower-middle and low income countries (Table 1). This was conducted in order to identify differences in monitoring methods and experiences of high-income countries relative to developing nations. Answers to Q5–8 were coded according to theme using qualitative thematic analysis. Core themes were predefined directly following Q5–8, and sub-themes then defined according to the answers to the questions. The same or very similar answers were assigned the same code and the numbers of countries providing the answers estimated for each code. The most frequently reported sub-themes were summarized in the results.

**Table 1:** Methods of monitoring of salt intake in countries participating in the survey, sorted by method and national income grouping*.

	Country	Type of survey	Specific method	Context of monitoring	Future monitoring planned	National income group
Dietary survey only	Canada	National nutrition and health survey	24 h food intake survey, adults and children, once only	National nutrition/health assessment & developing salt reduction initiative	Yes	High
	Cyprus	National nutrition and health survey	24 h food intake survey, adults and children, once only	National nutrition/health assessment & salt reduction initiative	Not known	High
	France	National nutrition and health survey	Food diary with weighing, adults and children by gender, repeated	National nutrition/health assessment	Yes	High
	Japan	National nutrition and health survey	Food diary with weighing, adults and children, repeated	Salt reduction initiative	No	High
	Kuwait	National nutrition and health survey	24 h food intake survey, adults, once only	National nutrition/health assessment	Not known	High
	Poland	Household budget survey	Amount of food purchased, all ages, once only	Salt reduction initiative	Yes	High
	South Korea	National nutrition and health survey	Nationally-representative 24 h food intake survey, adults and children, repeated	National nutrition/health assessment	Not known	High
	USA	National nutrition and health survey	24 h food intake survey, adults and children, repeated	National nutrition/health assessment and the development of a salt reduction initiative	Yes	High
	Costa Rica	National and sub-national nutrition and health surveys	Nationally-representative weekly-recall of salt added at table, by all household members, once only, plus 24 h recall in sub-population**	Iodine and flouride fortification program & the development of a salt reduction initiative	Yes	Upper-middle
	Malaysia	National nutrition and health survey	24 h food intake survey, adults, once only***	National nutrition/health assessment & the development of a salt reduction initiative	Yes	Upper middle
	Brazil [24]	National household budget survey	Amount of food purchased (repeated survey for all in household) and 24 h recall food intake survey (adults and adolescents, once only)	National nutrition/health assessment & the development of a salt reduction initiative	Yes	Upper-middle
	*Mexico*	*Research study*	*24 h food intake survey, adults, once only, for sub-population*	*Scientific concern about salt intakes*	*Yes*	*Upper-middle*
	*India * [*25*]	*Research study*	*Food frequency questionnaire, adults, once only, in sub-population*	*Scientific concern about salt intakes*	*Yes*	*Lower-middle*
	China	National household survey	24 h food intake measurements and food frequency questionnaire repeated every 10 years for all age groups	National nutrition/health assessment	Yes	Lower-middle
	Philippines	National household survey	Weighing household salt in national survey, repeated over time, applies to whole household	National nutrition/health assessment	Yes	Lower-middle
	Thailand	National household survey	Food frequency questionnaire, conducted once, adults only	Salt reduction initiative	Not known	Lower-middle
Dietary survey and 24 h urine nalaysis	Belgium	National nutrition and health survey (diet) and salt specific survey (urine)	National 24 h food intake survey (adults +15 yrs); 24 h urine for sub-population (adults), conducted once	Salt reduction initiative	Yes	High
	Finland [16]	National CVD/chronic disease risk factor assessment and national nutrition and health survey	National 24 h food intake survey and 24 h urine representative of 5 large areas (repeated)	Salt reduction initiative	Yes, in 2012	High
	Hungary [26]	National nutrition and health survey (diet) and salt specific survey (urine)	National food diary with weighing and 24 h urine in sub-population, repeated, adults and children	Salt reduction initiative	Yes	High
	Netherlands [27]	National nutrition and health survey and salt specific survey	24 h food intake survey for national (repeated every 4/5 yrs.); 24 h urine for sub-opulation (cohort), both adults and children	Salt reduction initiative	Yes	High
	Argentina [28]	National household budget survey (diet) and specific salt-survey (in sub-population)	Amount of food purchased for national survey and 24 h urine in sub-population, both repeated, all age groups	Salt reduction initiative	Yes	Upper middle
	Bulgaria [29]	National nutrition and health survey plus study of sub-population	24 h food intake survey for national; 24 h urine for subpopulation (both repeated)	National nutrition/health assessment	No	Upper middle
Dietary survey and spot urine	Australia [30]	National nutrition and health survey	24 h food intake survey (repeated) with spot urine (intention to repeat)	Salt reduction initiative and iodine fortification program	Yes	High
	Ireland [31]	National nutrition and health survey	Food diary (repeated) and spot urine (once), in adults	Salt reduction initiative	Yes	High
	New Zealand [32]	National nutrition and health survey	National 24 h food intake survey and spot urine, adults (+15 yrs), once only	National nutrition/health assessment	No	High
Spot urine analysis only	*Vietnam*	*Research study*	*Spot urine, adults, repeated pre-and post intervention only, in sub-population*	*Scientific concern about iodine deficiency*	*Yes*	*Lower-middle*
	*Bangladesh * [*33*]	*Research study*	*Spot urine, adults, once only, in sub-population*	*Scientific concern about high salt intakes*	*Yes*	*Low*
24 h urine only	Barbados	Salt-specific survey	Nationally representative 24 h urine, adults, once only	Salt reduction initiative	Yes (completion of survey)	High
	Italy	National CVD/chronic disease risk factor assessment and national nutrition and health survey	Nationally representative 24 h urine, adults and children, once only	Salt reduction initiative	Yes	High
	Singapore [34]	Salt-specific survey	Nationally representative 24 h urine, adults (18–79 yrs), to be repeated	National nutrition/health assessment	Yes	High
	Slovenia [35]	Salt-specific survey	Nationally representative 24 h urine, adults by gender, once only	Salt reduction initiative	Yes	High
	Spain [36]	Salt-specific survey	Nationally representative 24 h urine survey, adults, once	Salt reduction initiative	Not known	High
	Switzerland	Salt-specific survey	Nationally- representative 24 h urine survey, adults (+15), once	Salt reduction initiative	Yes	High
	UK [37]	Salt-specific survey	Nationally representative 24 h urine survey, adults, repeated	Salt reduction initiative	Not known	High
	*Iran*	*Research study*	*24 h urine analysis, adults, repeated, in sub-population*	*Scientific concern about salt intakes*	*No*	*Upper-middle*
	*South Africa * [*38*]	*Research study*	*24 h urine analysis, adults, once only in sub-population*	*Scientific concern about salt intakes*	*Not known*	*Upper-middle*

*Italicised countries conducted research studies only, not national monitoring.*

* References to results included where available.

** Spot urine analysis has also been conducted in Costa Rica, but for iodine and fluoride only.

*** In addition, a 24 h urine analysis was conducted in 2010 to obtain preliminary data of total salt intake in Malaysia.

## Results

### Responses

In total, 52 countries were contacted, and 45 responded to the survey (86%). Of the 45 respondents, 51% were high-income countries, 24% were upper-middle income countries, 20% were lower-middle income countries, and 1% were low income countries. The high proportion of responses from high-income countries was anticipated since that is where most salt monitoring activities are already in place. Six of the non-high income countries reported monitoring in the form of research studies rather than formal government monitoring. These countries were included in order to increase the number of developing countries in the sample, but the results analysed separately.

### Extent of monitoring

In the sample of 45 countries, 30 had already conducted national assessments of salt intake (Table 1). 21 of these 30 countries were planning to conduct further intake assessments in an upgraded form in the near future. In addition, independent scientific studies had been carried out by research organisations in sub samples of the population in six countries, four of which said that monitoring was planned for the future. (Table 1)

One further country – Chile –reported they had recently conducted a preliminary 24 hour urine study, but provide inadequate information. The eight remaining of the 45 countries reported no salt intake monitoring activities ongoing or in the past. One of these nine countries (Fiji) said they were planning to conduct intake monitoring in the future.

Of the 30 countries reporting formal national assessments of salt intake, 22 (73%) were high income countries. Of the remaining eight, five were upper-middle income and three were low-middle income. No low-income countries reported formal national assessments of salt intake. Of the countries reporting research-based studies of sub-populations, one was low-income, two low-middle income and three upper-middle income countries (Table 1). While these types of studies were not the focus of the survey, the results are considered here as they increase the input from low and middle income countries.

### Type of monitoring

The 30 countries either incorporated salt monitoring into national diet and nutrition surveys (n = 20), cardiovascular/chronic disease risk surveys (n = 2), stand-alone salt intake assessments (n = 9), or household expenditure surveys (n = 3). Four countries used more than one of these types of survey (Table 1).

Less than half of the 30 countries (n = 13) used 24 hour urinary analysis to assess salt intake. 11 of these were high income countries, and two were upper-middle income countries. The sample was only confirmed to be nationally representative in seven of these countries, all high- income. Three high income countries used spot urine tests (combined with dietary surveys). Five of the 21 countries planning upgraded assessments in the future specified they would be using 24 hour urine analysis, with one considering either 24 hour or spot urine.

23 of the 30 countries monitored salt intake through dietary surveys; nine of these countries also conducted urine analysis, meaning that 14 conducted household surveys alone. Surveys typically measured salt intake through dietary measurements (dietary recall, food frequency questionnaires, or food diaries/picture books with weighing), but three countries used expenditure measurements in household budget surveys.

Of the six countries which reported research studies, two of the upper-middle income countries used 24 hour urine and one used dietary surveys alone, as did one lower middle income country. The other low-middle income country used spot urine, as did the low income country in the survey.

### Experiences of the different monitoring methods

#### 24 hour urine analysis

Countries that used 24 hour urine analysis prioritised it as the “gold standard” for accuracy. But several of these countries reported that resource constraints meant they had to limit the sampling in some way. Spain, for example, used a small but representative sample, and the Netherlands a non-representative cohort. Both countries reported that, despite the limitations, it was better to use 24 hour urine in a more limited sample than not at all. Whilst still not gold-standard practice, this approach enabled them to obtain reasonably accurate information in a practical way, within the allocated resources.

Some countries that used 24 hour urine analysis reported they would consider alternative more practical methods in the future. In Singapore, there is interest in exploring other methods to estimate sodium intake as effectively as 24 hour urine collection. In Argentina, the need for more cost-effective and less logistically intensive ways to estimate salt intake was highlighted.

Three high-income countries decided against using 24 hour urine on the basis of the burdensome nature of collecting a nationally representative sample. Australia, for example, which is using a combination of dietary survey and spot urine measurements in its current survey, highlighted the impracticality of asking people to collect 24 hour urine samples with no supervision in such a large scale national survey. The rationale behind this was that 24 hour samples were best suited to smaller scientific studies undertaken by specialised researchers. Other countries also selected what they saw as more practical approaches than 24 hour urine. France used a food diary method to obtain dietary information and New Zealand opted for spot urines. Researchers from one low-income country (Bangladesh) highlighted the need for a simpler estimation method noting that 24 hour urine collection would be difficult in a large number of subjects.

Several countries considered the low response rate achieved in different surveys, including 24 hour urine collection, as a major issue. Switzerland reported a response rate of 10% and Spain a response rate of 25%. In the UK, the response rate to the 2005/06 nationally representative survey of adults was only 42.8%, despite participants being offered a £15 incentive. Iran reported having to use a “smaller sample than originally intended” in a regional survey because of low response rates. Switzerland also noted the extra challenges of obtaining responses from children and young people.

However, other countries had reasonably satisfactory response rates (Belgium at 80%, Italy at 40–75%, Singapore at 60–70%). The good response rate from Singapore was attributed to the provision of clear verbal and written instructions on the urine collection, and minimisation of inconvenience for the participants by asking field staff to retrieve the urine samples from them. Belgium cited the provision of €50 supermarket voucher incentives as a factor in success.

#### Spot urine tests

Of the five countries that reported using spot urine collections, Australia explained that it was because spot urines provided useful information at a population-wide level over time. Bangladesh indicated spot urines were “easily administrable and inexpensive” for a research study and provided an idea of population mean intake. Researchers in Vietnam also said they worked well, yielding a response rate of 97%. The countries that were using spot urines did so because they were felt to be more practical to undertake and easier to get an adequate response rate in comparison to 24 hour urines.

#### Dietary surveys

An on-going dietary or household budget survey was the method used by all the middle-income countries responding to the survey. In these cases, dietary or household budget surveys already existed, so salt intake was either already being measured or could be added with relatively few additional resources relative to urine sampling. Brazil had adopted this approach to make full use of existing data sources before gathering further data in future. However, the results from Poland that were based mainly on a household budget survey were thought to be “highly insufficient”.

Most high and middle income countries were reasonably satisfied with results from dietary surveys, but cited lack of data on discretionary use of salt in the home as a difficult challenge. In Hungary, for example, respondents failed to complete the question on discretionary salt intake accurately. In France, the estimate obtained from a dietary survey was thought to be very rough but there was doubt about whether it would have been possible to evaluate more accurately. Several countries noted that dietary surveys also placed a burden on participants and were subject to underreporting of food consumption.

The two countries most satisfied with the accuracy of their dietary survey results (Australia and Finland) said this was due to robust survey instruments from which salt intake could be calculated. Finland pointed out that the data on dietary salt intake was quite accurate compared to urine analysis *but* that it required a very high quality recipe-based food composition database to ensure that salt used during cooking is not missed. Finland and France both noted the difficulties of maintaining up-to-date food composition tables.

### Overall challenges and factors for success

Factors contributing to success in monitoring salt intakes emerged almost exclusively from high income countries (only three middle income countries responded to the question in the survey). The leading factor cited was the presence of sufficient skills and capacity, such as: the existence of national scientific experts and prior experience of carrying out large national surveys (Finland); scientific societies coming together (Italy); continuous Research and Development (R&D) investment and support from a government-affiliated research institute (South Korea); the presence of well-trained staff (Australia, Singapore); and the “right skills mix” (UK). Technical support from third-parties such as the European Salt Action Network and the WHO was also highlighted. In Hungary previous data collections (in this case on children in kindergartens showing high rates of intake) were viewed as critical for mobilizing interest in further monitoring.

The second factor for success was presence of sufficient financial resources. Singapore cited “adequate funds”, Switzerland reported “sufficient staffing and resources” and adequate resources were identified as crucial by the UK, Australia and Canada.

Thirdly, several high-income countries named political support as critical for success, (Netherlands, New Zealand, Spain, UK, US). Recognition by key federal agencies about the importance of the salt issue was highlighted as crucial in the US. In Europe, Hungary, Poland and Spain referred to the High Level Group on Nutrition and Physical Activity created by the European Commission as key in their efforts to obtain political support for monitoring. Political support was also highlighted as a necessary factor behind the financing of the salt monitoring work in the Netherlands.

The fourth critical factor for success was partnerships, co-operation and collaboration. Notably, the three middle income countries that responded to the question about factors for success all cited co-operation and collaboration with others as critical to efforts. Argentina, for example, highlighted support of NGOs and collaboration with the food industry as key factors in successful implementation of salt monitoring activities.

Fifth, policy and legal frameworks that require the monitoring of salt such as Healthy Japan 21 and regulations for warning labels on foods with high salt content in Finland were seen as key drivers.

The leading challenge named by all countries was financial. Several high income countries (e.g. Finland, France, Italy, Netherlands, Switzerland, and UK) noted how expensive monitoring was. Finding the money to finance the surveys (continuously) was highlighted as the hardest challenge in the Netherlands where both urine and food consumption surveys were deemed to be expensive. The cost of doing a nationwide urine survey was also seen as a barrier in the United States. In all low and middle income countries, cost was also highlighted as the leading challenge, either as a result of the lack of a dedicated budget or low resources in general. In Thailand, for example, it was noted that budgets tended to be directed towards health promotion campaigns rather than monitoring.

Apart from specific methodological challenges, the other leading challenge named by middle income countries was lack of capacity and political support. The lack of capacity in Thailand presented a real challenge to progress and a key challenge in Mexico had been to convince authorities of the need for the survey. Low prioritization in government was named as the primary challenge in Argentina.

## Discussion

Despite scientific concerns about lack of reliability of alternative methods for measuring sodium intake, only half of the high income countries and two of the upper-middle income countries in the survey used the gold standard of 24 hour urine analysis. Only just over half of these were confirmed to be nationally-representative samples. All the other high-and middle income countries used dietary survey methods, in three cases combined with spot urine. There were no national salt monitoring activities in low income countries.

These results reflect both the available resources and the view that the accuracy of 24 hour urine assessments in a nationally representative sample relative to other methods is not merited in view of the increased cost and burden of undertaking such assessments. Instead,many countries focused on generating what they perceived as adequate information to support policies and programs to address excess intake through dietary surveys, spot tests, and 24 hour urine on non-representative samples. The countries that did use 24 hour urine on nationally representative samples did so because they prioritised accuracy and representability of survey findings. The experiences of these countries suggest that successful monitoring using 24 hour urine analysis requires the presence of sufficient skills and capacity, financial resources and political support.

The results of this survey present a picture of the types and experiences of methods used in countries to monitor salt intake. However, the survey had some limitations. First, it is likely that not all countries undertaking salt monitoring activities at the time of the survey were included. 14% (n = 7) of countries contacted failed to respond and although it cannot be certain these countries were conducting monitoring, compared to the survey as a whole, low, lower-middle and upper-middle income were disproportionately represented among the non-respondents (n = 5, or 71% of all non-respondents, compared with 49% of the respondents sample). In addition, some countries may have been inadvertently excluded due to lack of information, which is more likely for low, lower-middle and upper-middle income countries (information from high-income countries being captured well through existing lists (18, 19)). Second, while all of the responding countries provided complete details about their monitoring methods (Q1–4), and most countries answered some questions about their experiences of implementation (Q5–8), 10 of the countries reporting either government monitoring or research studies did not complete *all* the questions on their perceptions of implementation. Though countries were requested to answer the questions, they stated that without broader consultation with colleagues, they did not feel able to report an official view given that the questions concerned perceptions of experiences. In addition, as for any qualitative study, the survey relied on the perceptions from respondent, which may not reflect a universal view.

Despite these limitations, the study has added considerably to what is known about the experiences of different countries in conducting salt monitoring activities and the lessons that can be learnt from these experiences. The main implications of the study are fourfold. First, there is a strong need for the scientific research community to develop more practical ways of conducting 24 hour urine assessments or identify ways to make alternative methods of assessment more accurate. Some work on this is already underway but the results need to be widely communicated to ensure that the countries considering salt monitoring in the near future can benefit from the new insights. [17], [22]

Second, governments need to design the most robust survey instruments that are feasible to monitor salt intake in the context of available resources and political support. Partnerships, collaborative efforts and the potential to build upon existing surveys should be used to support such efforts. Whilst this paper only covered the monitoring of salt intakes, leading sources of salt intake (e.g. salt added at table; condiments; processed foods) and consumer knowledge, attitudes and behaviour towards salt also need to be assessed, both to inform future salt reduction efforts, and to obtain a baseline from which to measure future progress.

Third, the WHO and regional organisations should increase the capacity of low and middle income countries to undertake monitoring by providing governments with increased technical support. The PAHO Protocol for Population Level Sodium Determination in 24 hour Urine Samples [23], for example, could be incorporated into regional training programs, additional technical forums for exchanging experience between countries organised and a global registry of monitoring activities established.

Fourth, the salt advocacy community should work together to identify potential sources of national and international funding while continuing efforts to build the political support. Funders should support research to strengthen monitoring. Given limitations of government funding, the scientific research community should seek such funding to undertake independent salt monitoring activities to compliment and strengthen political support for national programs.

Countries around the world are mobilising in their efforts to implement programs to reduce salt intake as a feasible cost-effective measure to reduce the burden of diet-related chronic diseases. Yet this survey revealed real challenges to effective and accurate monitoring of such programs. However, simple actions by governments, international agencies, NGOs and researchers could go a long way to overcome these challenges and ensure that the tangible benefits of national salt reduction programs can be fully realised and quantified.

## Supporting Information

Supplement S1 Written questionnaire used for the survey.
(DOC)
