# Supplementary material for: National Approaches to Monitoring Population Salt Intake: A Trade-Off between Accuracy and Practicality?
Source: PLoS One. 2012 Oct 17;7(10):e46727. doi: 10.1371/journal.pone.0046727 (PMC3474782; doi:10.1371/journal.pone.0046727)
Supplement: Supplement S1 — Written questionnaire used for the survey. (DOC) [file pone.0046727.s001.doc]

Supplement 1: Written questionnaire used for the survey

1. Is there a system in place in your country for:
   1. baseline assessment of salt intake, and/or; or
   2. monitoring the affects of salt reduction activities.
2. If you do have a system in place for 1a and/or 1b, we would appreciate it if you could provide more details in questions 3-9. If you do not, it would be useful if you could let us know if you have any plans to start monitoring activities over the next five years, and if so, what activities?
3. If you have a system in place for (1a) baseline assessment of salt intake, how do you conduct the assessment? We are specifically interested in:
   1. Whether the assessment is representative of the national population or of sub-groups
   2. The sample size and how the sample is generated
   3. Whether you use a dietary survey (weighed diary or 24 hour recall; inclusion of foods eaten away from home or not, etc) or urinary analysis (spot tests, 24hr urine collection)
   4. The response rates and what influences the response rates
   5. The dates and frequency of the assessments
   6. Whether the assessment is combined with assessments of other nutrients
   7. The costs involved
   8. Who is responsible for the assessment activities
4. If you have a system in place for (1b) monitoring the affects of salt reduction activities, have you collected information on effects on salt intake? If so, how do you conduct the assessment (see 3)?
5. If you have a system in place for salt monitoring for 1a and/or 1b, have the methods been successfully implemented? If so, what have been the three key factors in supporting successful implementation? Please specify for 1aand/or 1b, where applicable.
6. What have been the three main drawbacks and challenges of using these methods of assessment? Please specify for 1a and/or1b, where applicable.
7. Have you considered adopting alternative/additional methods of assessment and chosen not to do so? Or have you tried other methods of assessment in the past and ceased to use these methods? If so, why? Please specify for 1a and/or 1b, where applicable.
8. What are the three major lessons learned from your salt monitoring experiences? Please specify for 1a and/or1b, where applicable.
9. If you are able to pass on any results of your monitoring activities, it would be very much appreciated.

.
